# Mg, Zn Substituted Calcium Phosphates—Thermodynamic Modeling, Biomimetic Synthesis in the Presence of Low-Weight Amino Acids and High Temperature Properties

**DOI:** 10.3390/ma16206638

**Published:** 2023-10-11

**Authors:** Diana Rabadjieva, Rumiana Gergulova, Kostadinka Sezanova, Daniela Kovacheva, Rositsa Titorenkova

**Affiliations:** 1 Institute of General and Inorganic Chemistry, Bulgarian Academy of Sciences, Acad. G. Bonchev Str., bl. 11, 1113 Sofia, Bulgaria; rumigg@yahoo.com (R.G.); ksezanova@abv.bg (K.S.); didka@svr.igic.bas.bg (D.K.); 2 Institute of Mineralogy and Crystallography, Bulgarian Academy of Sciences, Acad. G. Bonchev Str., bl. 107, 1113 Sofia, Bulgaria; rositsatitorenkova@imc.bas.bg

**Keywords:** calcium phosphates, thermodynamic modeling, biomimetic synthesis, amino acids, complexation reaction, Rietveld analysis

## Abstract

The preparation of specially doped calcium phosphates (CaPs) is receiving a great deal of attention from researchers due to CaPs’ enhanced capabilities for application in medicine. Complexation and precipitation in a complicated electrolyte system including simulated body fluids that are enriched with Mg^2+^ and Zn^2+^ ions and modified with glycine, alanine and valine were first evaluated using a thermodynamic equilibrium model. The influence of the type and concentration of amino acid on the incorporation degree of Mg and Zn into the solid phases was predicted. Experimental studies, designed on the basis of thermodynamic calculations, confirmed the predictions. Amorphous calcium phosphates double-doped with Mg and Zn were biomimetically precipitated and transformed into Mg, Zn-β—tricalcium phosphates (TCP) upon calcination. The Rietveld refinement confirmed that Mg^2+^ and Zn^2+^ substituted Ca^2+^ only at the octahedral sites of β-TCP, and in some cases, fully displacing the Ca^2+^ from them. The resulting Mg, Zn-β–TCP can serve as a reservoir for Mg and Zn ions when included in the formulation of a biomaterial for bone remodeling. The research conducted reveals the effect of combining mathematical models with experimental studies to pre-evaluate the influence of various additives in the design of materials with predetermined properties.

## 1. Introduction

The biomimetic approach is used to obtain materials whose composition, structure and morphology resemble those of hard tissues. Simulation of the biomineralization process is most often reduced to the use of simulated body fluids (SBF) with a composition corresponding to the inorganic component of blood plasma [1,2]. Both the nucleation and growth of calcium phosphate crystals in natural biomineralization processes are controlled by organic molecules, mainly proteins and polysaccharides. Therefore, research on the synthesis of calcium phosphates in the presence of organic molecules is a promising trend for preparation of biomaterials applicable in dental and orthopedic medicine. Since amino acids (AA) are involved in the acidic domains of non-collagen proteins and are found in trace amounts as free amino acids in blood plasma, red blood cells and muscles, they are given special consideration [3]. The majority of studies on the subject focus on how amino acids affect the rate of conversion of primary precipitated amorphous calcium phosphate (ACP) into hydroxyapatite (HA) and the growth of the latter’s crystals [4,5,6,7,8,9,10,11,12].

The synthesis of specifically doped calcium phosphates attracts considerable attention because of the changes in their properties achieved in this way. It was recently shown that Zn-doped hydroxyapatite possesses better anti-bacterial properties and osteoblastic-proliferation activities [13]; Zn- or Ga-doped calcium phosphate coatings decreased corrosiveness of metal implants and enhanced cell affinity [14]; Mg-doped biphasic calcium phosphate nanoparticles containing silver revealed excellent cytocompatibility with a culture of human bone marrow-derived mesenchymal stem cells [15], etc. Mg and Zn are preferred over all other substituents because they are crucial in the formation of the skeleton [16,17]. Their incorporation in a wide concentration range in the composition of calcium phosphate materials does not alter the biocompatibility of the latter [17,18].

There are no data on the effect of amino acids on the composition and morphology of ion-substituted calcium phosphates despite the fact that ion-substituted calcium orthophosphates build up the inorganic component of the hard tissues.

Mathematical models are an effective tool to predict ongoing processes under varying conditions. Their main advantage is that they take into account the simultaneous influence of multiple factors in the process of obtaining materials with predetermined properties and thus to facilitate experimental research or to optimize material applications [19,20]. The models based on the principle of the chemical thermodynamics are usually employed to determine the concentration range at which stable or metastable crystallization of solid phases occurs, and as a result, to predict system behavior with changes of the temperature, component concentrations, etc. [21,22].

Conducting systematic studies on the effect of type and concentration of AAs over a wide range would be a long and complex process. In our previous studies [23,24], we have shown the possibility of using a mathematical approach based on chemical thermodynamics to predict and elucidate the influence of the inorganic composition of the simulated body fluids on the formation and transformation of amorphous calcium phosphate and dicalcium phosphate dihydrate. Based on the results of the thermodynamic calculations, targeted experimental studies were carried out that confirmed the predictions made. An analogous approach was applied in the present work, in which the thermodynamic modeling was complicated by the presence of AAs. This affects the complexation reactions with the metal cations in SBF.

The aim of this work is to predict and experimentally confirm the synthesis of doubly-doped (Mg and Zn) calcium phosphates by thermodynamic modeling of the complexation and precipitation processes in simulated body fluid (SBF) solutions enriched with various low-weight amino acids. To this purpose, the systems MCl_2_—AA—SBF– H_2_O (M = Ca, Mg, Zn; AA = Gly, Ala, Val; SBF includes Na^+^, K^+^, Ca^2+^, Mg^2+^, HPO_4_^2−^, HCO_3_^−^, Cl^−^, and SO_4_^2−^ ions) were modeled. On this basis, experimental studies were carried out with SBFs containing three amino acids, glycine (Gly), alanine (Ala) and valine (Val), using two concentrations in diluted and concentrated ranges. The influence of amino acids on the composition and morphology of freshly precipitated amorphous calcium phosphate was monitored, and its phase transformations during high-temperature treatment (400–1000 °C) were followed by applying chemical, IR, TEM, BET, XRD analyses and Rietveld refinement.

## 2. Materials and Methods

### 2.1. Thermodynamic Calculations

The MCl_2_—AA—SBF– H_2_O systems (M = Ca, Mg, Zn; AA = Gly, Ala, Val; SBF includes Na^+^, K^+^, Ca^2+^, Mg^2+^, HPO_4_^2−^, HCO_3_^−^, Cl^−^, SO_4_^2−^) were thermodynamically modeled and species distributions in the solutions together with saturation indices of possible solid phases were calculated.

The computer program PHREEQCI, v. 2.14.3 [25], which is based on an ion-association model, was used. The ion-association model is a chemical equilibrium model which takes into account weak interactions between ions with opposing charges to create ion pairs (associates) in solution or solid phases. Ion association and salt crystallization reactions are specified by a mass-action expression with suitable formation constants, in which the extended Debye–Hückel theory is used to compute the activity coefficients of all potential species.

An extended thermodynamic database with thermodynamic formation constants (logK) from NIST databases [26] was used in the calculations (Table S1, Supplementary Material). Three solubility products and ten complex formation constants were additionally included by us (Table 1).

**Figure 1:**
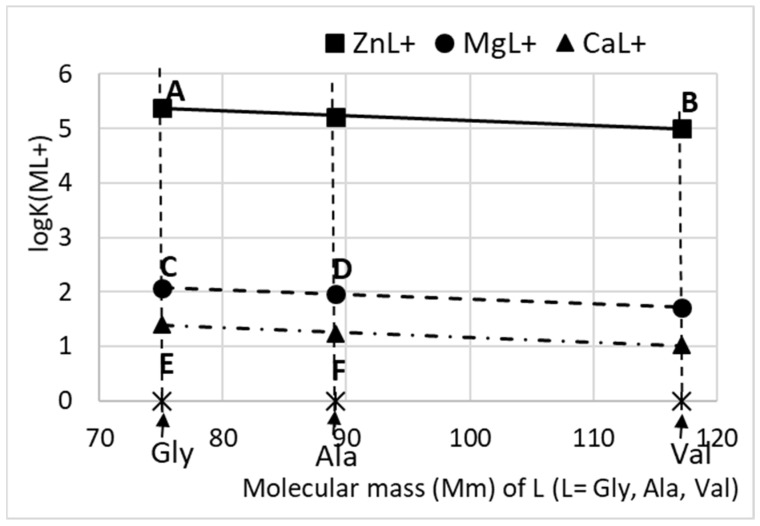
Thermodynamic formation constants (logK) of ML+ complexes. M = Mg, Ca, Zn; L = Gly, Ala, Val. The amino acids were presented with their molecular masses on the abscissa.

The logK values for alanine species MAla^+^ (M = Ca, Mg, Zn) at zero ionic strength and for valine species ZnVal^+^ at ionic strength of 0.01 (closest to 0) were taken from Sovago et al. [28]. The logK values for CaVal^+^ and MgVal^+^ were calculated in this study through translation of line AB (Figure 1) to the values of logK for MgGly^+^—MgAla^+^ (line CD) and CaGly^+^—CaAla^+^ (line EF), respectively. Our calculations considered only 1:1 complexes because experimental data indicated the formation of these complexes in aqueous solutions [29].

The concentrations of ions in Solution 2 and Solution 3 (Table 2), along with varying concentrations of amino acids, were used as input data for calculation of chemical species distribution. The solubility of AAs at 25 °C determines their concentrations range, namely: from 0 to 200 g L^−1^ for Gly (solubility at 25 °C 249.9 g L^−1^ [30]); from 0 to 100 g L^−1^ for Ala (solubility at 25 °C 166.5 g L^−1^ [30]); from 0 to 50 g L^−1^ for Val (solubility at 25 °C 88.5 g L^−1^ [30])

Additionally, Solution 1 and the buffer solution (Table 2), as well as a solution of 0.05 M KOH, were included in the modeling, simulating the precipitation process (as it is described in Section 2.2.2) for calculation of saturation indices of possible solid phases.

The saturation indices (SI) (Equation (1)) were calculated as indicators for possible salts precipitation:

SI = lg (IAP/K)(1)

where IAP is an ion activity product, and K is a solubility product.

From 24 solid phases having SI > 0 in the examined systems, only 15 were included in the model, namely CaCO_3_, CaHPO_4_·2H_2_O, Ca_3_(PO_4_) (amorphous), Ca_8_H_2_(PO_4_)_6_·5H_2_O, Ca_9_Mg(HPO_4_)(PO_4_)_6_, Ca_5_(PO_4_)_3_(OH), KMgPO_4_·6H_2_O, MgHPO_4_·3H_2_O, Mg_3_(PO_4_)_2_.22H_2_O, Mg_3_(PO_4_)_2_, Mg_3_(PO_4_)_2_·8H_2_O, Zn(OH)_2_, ZnCO_3_, ZnCO_3_·H_2_O and Zn_3_(PO_4_)_2_·4H_2_O. Phases with a negative SI or with a positive SI but no expected spontaneous crystallization were excluded from the model because they are high-temperature phases (β-Ca_3_(PO_4_)_2_, CaHPO_4_ and Ca_4_(PO_4_)_2_O), long-term maturated phases (CaMg(CO_3_)_2_, CaMg_3_(CO_3_)_4_, Zn_2_(OH)_3_Cl, Zn_4_(OH)_6_SO_4_, Zn_5_(OH)_8_Cl_2_), or the result of solution oxidation (ZnO). Ca_9_Mg(HPO_4_)(PO_4_)_6_ was used as an example of Mg-doped calcium phosphate for which the thermodynamic precipitation constant is known. Double-doped calcium phosphates were not included in the model because of a lack of data.

### 2.2. Biomimetic Synthesis of (Mg, Zn)-Doped Calcium Phosphates

#### 2.2.1. Initial Solutions

The synthesis of calcium phosphates in this work involved modified simulated body fluids. They were all created using a conventional simulated body fluid (SBFc) [26] (Table 2). K_2_HPO_4_ (Merck, Darmstatd, Germany, A.R.) was dissolved in modified calcium-free conventional simulated body fluid (Solution 1, Table 2), whereas CaCl_2_·2H_2_O (Sigma-Aldrich, St. Louis, MO, USA, A.R.), MgCl_2_·H_2_O (Merck, Darmstatd, Germany, A.R.), and ZnCl_2_·2H_2_O (Merck, Darmstatd, Germany, A.R.) were dissolved in modified phosphorus-free conventional simulated body fluid (Solution 2 and Solution 3, Table 2). In this way, preliminary precipitation was avoided.

The modified simulated bodily fluids (Table 2) were all made by mixing salt solutions that had already been prepared, namely: 1.37 M NaCl (INEOS, A.R., London, UK), 0.04 M NaHCO_3_ (SOLVAY, A.R., Brussels, Belgium), 0.03 M KCl (Merck, Darmstatd, Germany, A.R.), 0.01 M K_2_HPO_4_·(Merck, Darmstatd, Germany, A.R.), 0.015 M MgCl_2_·6H_2_O, 0.025 M CaCl_2_·2H_2_O, 0.005 M Na_2_SO_4_ (JLC-CHEMIE Hendel GmbH, Wohlen bei Bern, Switzerland, A.R.). The calculated amounts of K_2_HPO_4_·3H_2_O, CaCl_2_·2H_2_O, MgCl_2_·6H_2_O and ZnCl_2_·6H_2_O and the corresponding amounts of Gly (Sigma-Aldrich, St. Louis, MO, USA, A.R.), Ala (Sigma-Aldrich, St. Louis, MO, USA, A.R.) or Val (Sigma-Aldrich, St. Louis, MO, USA, A.R.) were used to prepare calcium- and phosphorus-free simulated body fluids. The concentrations of the AAs were selected to represent diluted solutions (7.5 g L^−1^) and concentrated solution regions (220 g L^−1^ Gly, 142 g L^−1^ Ala and 60 g L^−1^ Val) of AAs. In the second case, the aim was for the concentrations to be as close as possible to solubility at 25 °C but not to cause solid phase crystallization at room temperature (18–28 °C).

The pH of Solutions 1 and 2 was adjusted to 8.0–8.2 using 0.1 M HCl (Merck, Darmstatd, Germany, A.R.) or 0.05 M tris (hydroxymethyl) aminomethane (Sigma-Aldrich, St. Louis, MO, USA, A.R.). To prevent hydrolysis processes, the pH of Solution 3 was not adjusted.

#### 2.2.2. Precipitation Method

Solution 1, Solution 2 and Solution 3 (Table 2) were appended to matching buffer solutions having Gly, Ala or Val, with a velocity of 3 Ml min^−1^. pH of 8.0–8.2 was maintained with 0.05 M KOH. Combined apparatus for automatic titration and controlled synthesis (Titrando 907, Methrom AG) was used.

Two sets of experiments were carried out:

Series A—with concentration of amino acids Gly/Ala/Val of 7.5 g L^−1^, whose products will be hereinafter referred to as CPGly7, CPAla7 and CPVal7, respectively. Series A represents diluted solutions of AA.

Series B—with concentrated solutions of amino acids, namely 220 g L^−1^ of Gly, 142 g L^−1^ of Ala and 60 g L^−1^ of Val, whose products will be hereinafter referred to as CPGly220, CPAla142 and CPVal60, respectively.

Concentrations of Mg^2+^, Zn^2+^, Ca^2+^ and PO_4_^3−^ ions were calculated to produce calcium phosphate precursors with ratios Mg^2+^/Σ(M^2+^) = 7 mol%, Zn^2+^/Σ(M^2+^) = 3 mol% and Σ(M^2+^)/P = 1.67 (M = Ca, Mg, Zn), respectively. Then, the concentration of Mg^2+^ was doubled in order to provide the desired percentage of magnesium ions in the structure of the precursor [32]. The concentrations of Mg^2+^ and Zn^2+^ ions were selected on the basis of our previous studies on the biocompatibility of Mg- or Zn-modified tri-calcium phosphates [18].

The suspensions were left in the mother liquor for one hour while being constantly stirred at room temperature. They were then freeze-dried after being cleaned with water numerous times through decantation.

### 2.3. Calcination of (Mg, Zn)-Doped Calcium Phosphates

The dry precursors were calcined at 200, 400, 600, 800 and 1000 °C and atmospheric pressure. The powders were heated at a rate of 3 °C/min up to the desired temperature, which was kept constant for 3 h. A high-temperature furnace, type VP 04/17 of LAC Ltd. Company (Tokyo, Japan), was used.

### 2.4. Characterization

#### 2.4.1. Chemical Analysis

Complexometric analysis using EDTA and the indicator eriochrome black T at pH 10 was used to determine the total amount of Ca^2+^, Zn^2+^ and Mg^2+^ ions in the liquid and dissolved solid phases. The concentrations of Zn^2+^ and Mg^2+^ ions were determined by ICP-OES (PRODIGY 7, Teledyne, Leeman Labs, Hudson, NH, USA). Spectrophotometer NOVA 60 and Merck Spectroquant test kits were used for determination of the concentrations of PO_4_^3−^ and Cl^−^ ions. Each experiment is the result of three parallel samples. Analytical results are the mean values of three parallel measurements (*n* = 3) of each sample, i.e., RSD = 0.2–0.5% of complexomeric determination, RSD = 1–2% of spectrophotometric determination and RSD = 0.5–1% for ICP-OES measurements.

#### 2.4.2. Infrared Spectroscopy (IR)

Infrared spectra in transmission mode were measured on standard KBr pellets on a FT-IR spectrometer (Tensor 37, Bruker, Karlsruhe, Germany). The spectrometer was a grant to the Institute of mineralogy and crystallography, BAS from the Alexander von Humboldt Foundation (Berlin, Germany) in 2005.The operating parameters were a spectral range of 400–4000 cm^–1^ and a spectral resolution of 4 cm^–1^. The spectra were evaluated using OPUS 6.5; they were smoothed at 5 points, normalized to the matching maximum intensity, and baseline-corrected using rubber band correction.

#### 2.4.3. X-ray Diffraction Analysis (XRD)

A Bruker D8 Advance diffractometer (Bruker AXS Advanced X-ray Solutions GmbH, Karlsruhe, Germany) was used to perform powder XRD. The X-ray source was a Cu tube (λ = 1.5418 Å). The pattern record was made by a LynxEye detector (Bruker AXS Advanced X-ray Solutions GmbH, Karlsruhe, Germany). The data were gathered in the 10 to 90° 2θ range with a step of 0.03° 2θ and a counting rate of 57 s/step for the phase identification. With the aid of the ICDD-PDF2 (2014) database and Diffracplus EVA software(v. 4, 2014), the phase composition was determined. In the examined samples, whitlockite (Ca_3_(PO_4_)_2_—PDF # 09-0169), Ca_19_Zn_2_(PO_4_)_14_—PDF # 48-1196, and Ca_2.81_Mg_0.19_(PO_4_)_2_—PDF # 70-0682 were the principal calcium phosphate phases.

For the purpose of Rietveld structure refinement, the collection of diffraction patterns has the following parameters: room temperature; 2θ range—5 to 120° 2θ; 2θ step −0.02° 2θ; counting time per step—175 s, sample rotation—15 rpm. The Bruker Topas v. 4.2 program was used to carry out the Rietveld refinement [33]. Yashima et al. [34] crystal structure was used as the initial model for the refining.

The parameters varied in the calculations were as follows: (i) unit cell parameters were refined at zero shift, scale factor; (ii) a sixth order Chebyshev polynomial function was used to refine the background; (iii) occupancies of the Ca(1), Ca(2) and Ca(3) positions were kept fixed because their variation led to values within the experimental error; (iv) the occupancies of the Ca(4) and Ca(5) positions were varied, with the sum of the Ca, Mg and Zn ion occupancy values constrained to 0.5 for the Ca(4) position and to 1 for the Ca(5) position; (v) Ca_2_P_2_O_7_ was also included in the refinement.

#### 2.4.4. Transition Electron Microscopy (TEM) Analysis

Transmission electron microscope, apparatus JEOL TEM JEM-2100, Tokyo, Japan was used to analyze the morphology of the acquired precursors. Samples were prepared by dispersing the powders in water and sonicating for 1 min. The suspensions were dropped on standard carbon-copper grids.

#### 2.4.5. Measurement of Specific Surface Area

Low-temperature (77.4 K) N_2_ adsorption was applied to obtain the specific surface area (SSA) using a Quantachrome Instruments NOVA 1200e (USA). The Brunauer–Emmett–Teller (BET) theory was applied by the calculations.

## 3. Results

### 3.1. Thermodynamic Modeling

**Figure 2:**
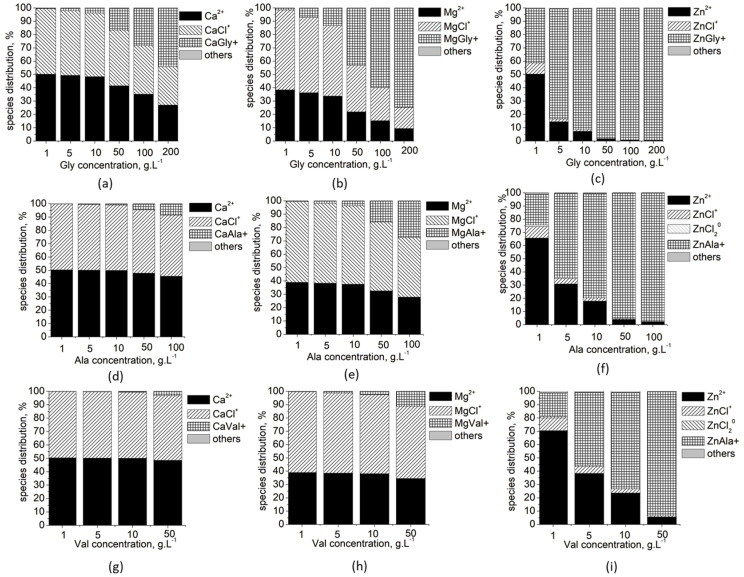
Species distribution of: Ca^2+^ ions in solution with varying concentration of Gly (**a**), Ala (**d**) and Val (**g**); Mg^2+^ ions in solution with varying concentration of Gly (**b**), Ala (**e**) and Val (**h**); and Zn^2+^ ions in solution with varying concentration of Gly (**c**), Ala (**f**) and Val (**i**).

Thermodynamic modeling of chemical species distribution (Figure 2) in the studied system shows that only three metal species dominate in all solutions at different concentrations: free M^2+^ ions, MCl^+^ and ML^+^ species. For low concentrations of Ala and Val, there are also ZnCl_2_^0^ species in amounts of up to 1–2%.

**Figure 3:**
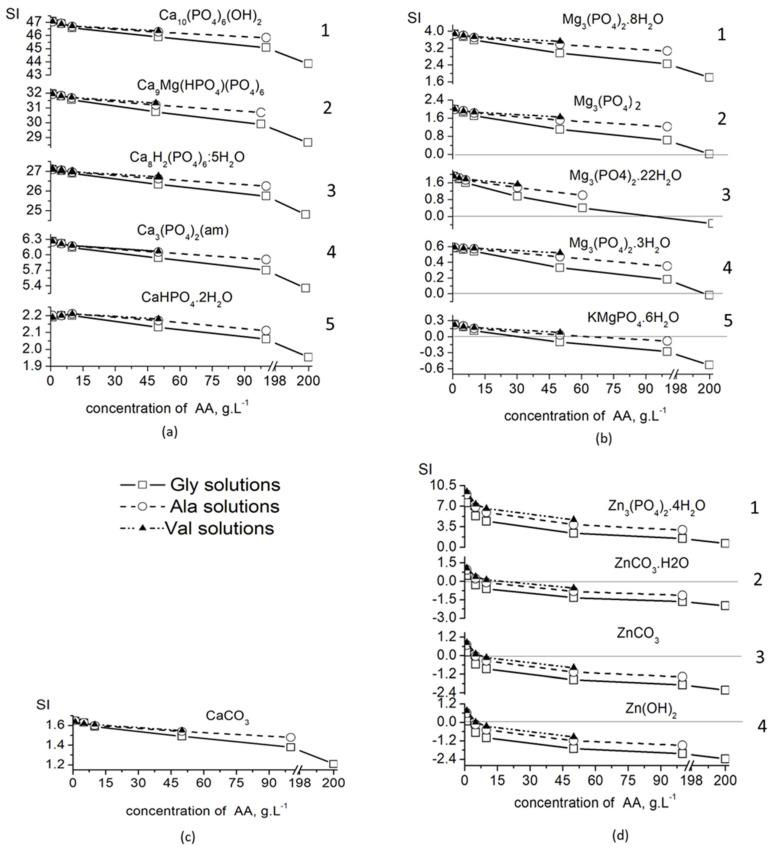
Changes of the saturation indices (SI) of possible-to-crystallize solid phases with increased the amino acid concentrations: (**a**) calcium salts 1—Ca_10_(PO_4_)_6_(OH)_2_; 2—Ca_9_Mg(HPO_4_)(PO_4_)_6_; 3—Ca_8_H_2_(PO_4_)_6_·5H_2_O; 4—Ca_3_(PO_4_)_2_(am); 5—CaHPO_4_·2H_2_O; (**b**) magnesium salts: 1—Mg_3_(PO_4_)_2_·8H_2_O; 2—Mg_3_(PO_4_)_2_; 4—Mg_3_(PO_4_)_2_·3H_2_O; 5—KMgPO_4_·6H_2_O (**c**) CaCO_3_; and (**d**) zinc salts: 1—Zn_3_(PO_4_)_2_·4H_2_O; 2—ZnCO_3_·2H_2_O; 3—ZnCO_3_; 4—Zn(OH)_2_. A straight line at SI = 0, separating the diluted from saturated solutions, is added where possible.

The calculated values of the saturation indices of the solid phases (Figure 3) show that, for the entire concentration range of the three amino acids, they are highest in the calcium phosphate phases (Figure 3a), and lowest in the zinc compounds (Figure 3d), of which only Zn_3_(PO_4_)_2_·4H_2_O (SI > 0) is expected to precipitate.

The magnesium solid phases take an intermediate position, and only Mg_3_(PO_4_)_2_·8H_2_O has a positive SI (Figure 3c). KMgPO_4_·6H_2_O (SI < 0) does not co-precipitate in the concentrated solutions of the amino acids, while Mg_3_(PO_4_)_2_, Mg_3_(PO_4_)_2_·3H_2_O and Mg_3_(PO_4_)_2_·22H_2_O co-precipitate in concentrated solutions of Val and Ala, but not of Gly.

The solutions remain supersaturated (SI > 0) with respect to all phases of calcium phosphate, including CaCO_3_, in the entire concentration range (Figure 3a,b), which means that, from a thermodynamic point of view, their precipitation is possible in all investigated solutions.

### 3.2. Biomimetic Synthesis

Based on the conclusions drawn from the thermodynamic modeling, two sets of experiments were carried out with different concentrations of amino acids: Series A, with concentration of Gly/Ala/Val of 7.5 g L^−1^, and Series B, with concentrated solutions of amino acids, namely 220 g L^−1^ of Gly, 142 g L^−1^ of Ala and 60 g L^−1^ of Val.

The results from chemical analysis are shown in Table 3 and reveal that substances with (Ca^2+^ + Mg^2+^ + Zn^2+^)/P ratio between 1.47 and 1.56 and content of Mg^2+^ ions between 5.86 and 8.09 m0l% and Zn^2+^ ions between 0.71 and 2.81 mol% were observed.

**Figure 4:**
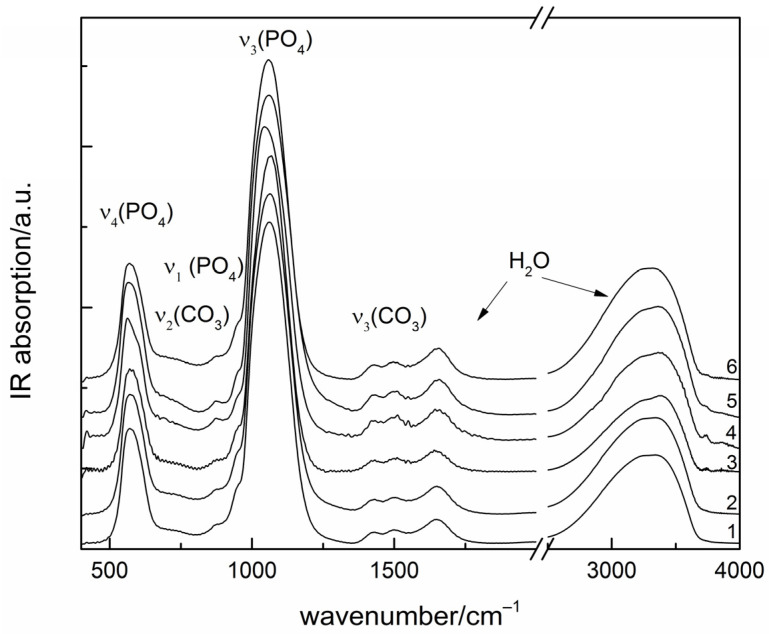
IR spectra of as precipitated calcium phosphates: 1—CPGly7; 2—CPAla7; 3—CPVal7; 4—CPGly220; 5—CPAla142; 6—CPVal60.

Regardless of the type and concentration of amino acids used, amorphous calcium phosphates were precipitated (Figure 4).

All the IR spectra are typical for amorphous calcium phosphate (ACP), with a broad absorption band of phosphate antisymmetric stretching centered near 1060 cm^−1^ and other phosphate peaks at 575 (ν_4_) and 950 (ν_1_) cm^−1^. Peaks at 1650 and around 3300 cm^−1^ are due to water molecules. Other weak absorption peaks at 1425, 1495 and 870 cm^−1^ are in the range of carbonate stretching and bending vibrations, respectively.

**Figure 5:**
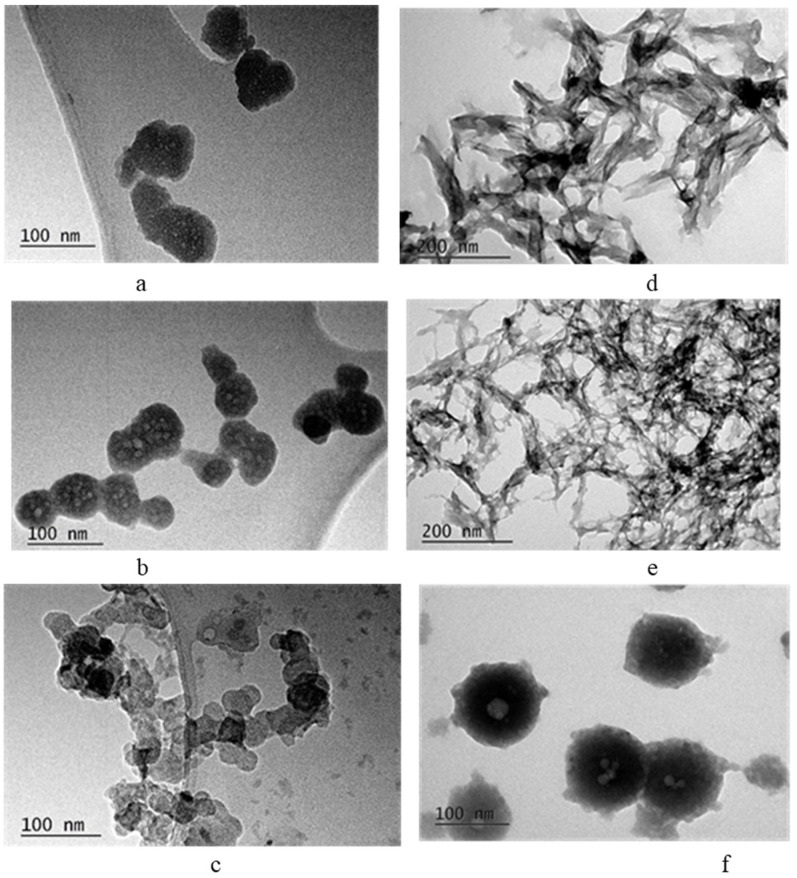
TEM images of CPGly7 (**a**), CPAla7 (**b**), CPVal7 (**c**), CPGly220 (**d**), CPAla142 (**e**) and CPVal60 (**f**).

TEM images (Figure 5) show, the particles of samples from Series A are spherical, with a diameter of about 50 nm in the case of Gly and Ala as additives and about 30 nm in the case of Val (Figure 5a–c). The shape of the particles in Series B that have Val as an additive is preserved, but the diameter increases to 100 nm (Figure 5f). In the case of Gly and Ala, the particles lose their spherical shape and elongate along one axis (Figure 5d,e).

The radical change in particle shape leads to an eight-fold increase in specific surface area (Table 4).

### 3.3. High Temperature Properties

**Figure 6:**
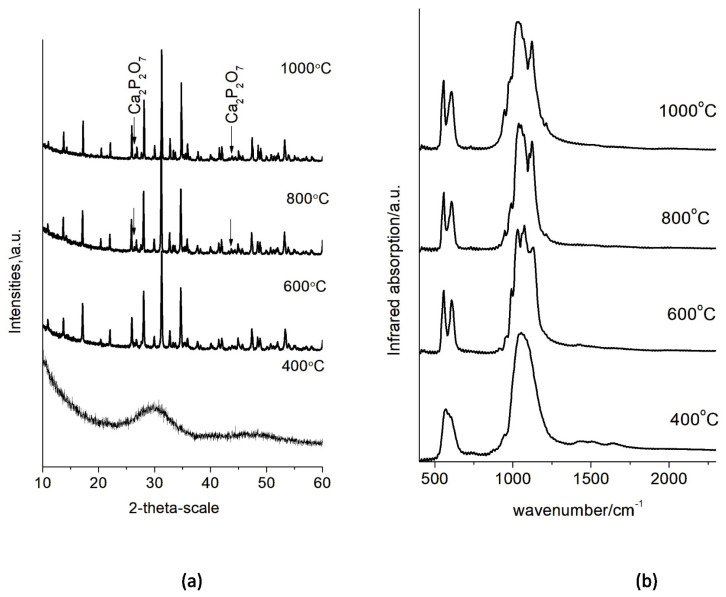
XRD powder patterns and IR spectra of selected calcium phosphate precursors calcined at different temperatures: (**a**) XRD powder patterns of CPVal7 (unmarked peaks—Mg, Zn-β-TCP) and (**b**) IR spectra of CPGly220.

The high-temperature phase transformations of the obtained calcium phosphate precursors were monitored by step-wise heating at 400, 600, 800 and 1000 °C. The IR and XRD analyses of the calcined samples revealed the transformation of the ion-modified amorphous calcium phosphate into (Mg, Zn)-containing β-tricalcium phosphate ((Mg, Zn)-β-TCP). Since all diffraction patterns and IR spectra were of the same type, only the XRD pattern of CPVal7 (Figure 6a) and the IR spectrum of CPGly220 (Figure 6b) are shown as examples.

At 400 °C all samples were still amorphous, but after heating at 600 °C for 3 h, crystalline (Mg, Zn)-β-TCP was obtained. At 800 and 1000 °C, the phase Ca_2_P_2_O_7_ appeared (Figure 6a). In the IR spectra, it was connected with the peaks at 735 and 1200 cm^−1^ characteristic for a P_2_O_7_ group (Figure 6b). The formation of Ca_2_P_2_O_7_ is a result of the thermal decomposition of the HPO_4_^−^ ions present in the hydrate shell of the amorphous particles.

## 4. Discussion

A useful tool for calculating the species distribution of the elements in the solution and the type and concentration interval of stable or metastable crystallization of solid phases is thermodynamic modeling, which is based on quantitative relationships between the concentration of the solutions and thermodynamic parameters, such as chemical potential, solubility product, equilibrium complexation constants, etc. In this work, a thermodynamic modeling approach for the systems MCl_2_—AA—SBF– H_2_O (M = Ca, Mg, Zn; AA = Gly, Ala, Val; SBF includes Na^+^, K^+^, Ca^2+^, Mg^2+^, HPO_4_^2−^, HCO_3_^−^, Cl^−^, and SO_4_^2−^ ions) is applied in order to predict incorporation of Mg^2+^ and Zn^2+^ ions in the solids depending on the type and concentration of the AAs used. On this basis, only two series of experimental studies were carried out, with SBFs enriched with Mg^2+^ and Zn^2+^ ions and containing three amino acids, glycine (Gly), alanine (Ala) and valine (Val), and using two concentrations in the diluted (7.5 g L^−1^) and concentrated (200 g L^−1^ Gly, 100 g L^−1^ Ala and 50 g L^−1^ Val) ranges. The differences in concentrations of the concentrated solutions are due to different solubility of the amino acids used.

For this purpose, the unknown stability constants of MgVal+ and CaVal+ were first determined in this study by approximating the known ones (Figure 1). The obtained values were included in database, and the calculations of Ca, Mg and Zn specie distribution (Figure 2) show that complexation in the solutions increases in the order Ca < Mg < Zn and Val < Ala < Gly, as well as with increasing amino acid concentration. As a result, the amounts of free ions in the solution decrease, which reduces the possibility that precipitation processes will take place and that Mg^2+^ and Zn^2+^ ions will be included in the composition of the solid phase. Thus, it is to be expected that, in concentrated solutions of Gly, the inclusion of Mg^2+^ and Zn^2+^ ions in the precipitates would be minimal and would increase in the concentrated solutions of Ala and Val. The calculated SI (Figure 3) show that, in dilute solutions of AA (up to 10 g L^−1^), the values of SI are close for all calcium, magnesium and zinc phases, which means that no effect of the type of amino acid on the degree of incorporation of Mg^2+^ and Zn^2+^ ions into the precipitate is to be expected in this case.

Experimental studies were designed on the basis of thermodynamic studies, and the results from the chemical analysis (Table 3) prove the predictions made. In the case of Series A, the concentrations of Mg^2+^ and Zn2+ ions for the three amino acids were similar. A slight trend to increase the content of Mg^2+^ and Zn^2+^ ions was observed in the sequence Gly → Ala → Val. This trend was more pronounced when concentrated acid solutions (Series B) were used (Table 3). The lowest concentrations of Mg^2+^ and Zn^2+^ ions were determined in the solid phase precipitated in a concentrated solution of Gly, and the highest concentrations were in valine.

The precipitation of amorphous calcium phosphates is in accordance with the dependence we previously discovered [23,24], which states that kinetic conditions, as opposed to thermodynamic ones, control the precipitation of ACP instead of the thermodynamically stable HA. During synthesis, upon addition of a solution containing HPO_4_^2−^ ions (Solution 1, Table 2), the latter will bind to the free Ca^2+^ ions, forming so-called Posner clusters at pH 8. The latter are the structural units that construct ACP [35]. The highest SI and thermodynamic stability are displayed by hydroxyapatite (Ca_10_(PO_4_)_6_(OH)_2_) followed by whitlockite (Ca_9_Mg(HPO_4_)(PO_4_)_6_). However, it is to be expected that the salt, whose structural units are already formed in the solution, will be the first to precipitate. Thus, quick kinetics and high pH favor the formation of ACP. As the ionic radii and electrical charge of Mg^2+^ and Zn^2+^ ions are close to those of Ca^2+^ ions, they are easily incorporated in the calcium phosphate structure, especially in dilute solutions where they exist as free ions in relatively high concentrations.

The concentration of amino acid influences the size and shape of primary precipitated particles and thus affects their specific surface area (Figure 5 and Table 4). Transformation from spherical to needle-shaped particles is connected with phase transformation from amorphous to poorly crystalline hydroxyapatite [6,7,9]. In our study, we did not obtain poorly crystalline hydroxyapatite. The samples remained X-ray amorphous even after calcination at 400 °C (Figure 6a,b). This is due to the presence of Mg^2+^ and Zn^2+^ ions that stabilize the amorphous phase and delay transformation to hydroxyapatite. We suppose that organic molecules with a linear carbon chain (Gly and Ala) promote the elongation of the spherical particles (Figure 5d,e), while a branched carbon chain (Val) stimulates their enlargement (Figure 5f).

High temperature treatment reveal formation of double-doped (Mg, Zn)-β-TCP (Figure 6). The Rietveld refinement was used in this study following the cation distribution in the structure of the double-substituted phases (calcined at 1000 °C).

The substitution of Mg^2+^ and Zn^2+^ for Ca^2+^ in the β-TCP structure was confirmed by the decrease in unit cell parameters in comparison with the unsubstituted β-TCP (Table 5). The decrease in the unit cell parameters *a* and *c* and in the volume V is proportional to the increase in the sum of concentrations of the substituting ions.

**Figure 7:**
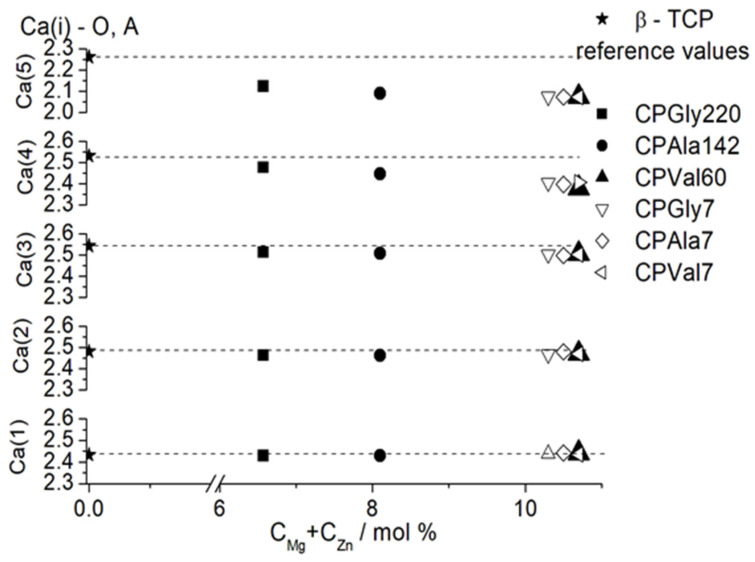
Dependence of the Ca(i)—O (i = 1–5) average distances on the sum of concentrations of substituting ions. Reference data are from Yashima et al. [34].

Comparison of the calculated average distances around the five Ca positions shows that distances around Ca(1) and Ca(2) positions remain almost unchanged for all substituted samples obtained at different concentrations of amino acids as compared to unsubstituted β-TCP (Figure 7). A slight decrease in the average distance is observed for Ca(3), while distances around Ca(4) and especially around Ca(5) drastically decrease in comparison with the values for the unsubstituted samples.

The calculated occupancy of Ca(4) and Ca(5) positions (Table 6) shows that both Mg and Zn ions have a tendency to predominantly occupy octahedral Ca(5) positions, in some cases fully displacing the Ca ions (sample CPVal7). Ca(4) position seems to be less preferable for small ions, and Ca^2+^ ions in that position are substituted by smaller amounts of Mg^2+^ and Zn^2+^ ions.

## 5. Conclusions

Mathematical models and experimental studies were combined in this research to pre-evaluate the impact of different additives on the design of materials applicable in medicine with predetermined attributes. Based on thermodynamic modeling of the ongoing processes in the systems MCl_2_—AA—SBF—H_2_O (M = Ca, Mg, Zn; AA = Gly, Ala, Val; SBF includes Na^+^, K^+^, Ca^2+^, Mg^2+^, HPO_4_^2−^, HCO_3_^−^, Cl^−^, SO_4_^2−^), the degree of inclusion of Mg and Zn in the probable solid phases was predicted depending on the amount of amino acids added. It was calculated that dilute amino acid solutions did not affect the incorporation of Mg and Zn into the solid phases, while concentrated ones did, i.e., the lowest concentrations were predicted in the presence of glycine, but the highest were predicted in valine. To fulfill the calculations, the unknown stability constants of MgVal+ and CaVal+ were first determined in this study by approximating the known ones.

Experimental studies are focused in only the two concentration regions of amino acids that give the most promising results, namely: 7.5 g L^−1^, representing the range of dilute solutions, and 60 g L^−1^ of Val, 142 g L^−1^ of Ala and 220 g L^−1^ of Gly, closed to saturation at 25 °C solution for each amino acid. The experiments verify the thermodynamic calculations. The content of Mg^2+^ and Zn^2+^ ions in the precipitated amorphous phase varies within narrow limits when the synthesis proceeds in dilute solution of amino acids. A strong tendency toward an increase in the content of Mg^2+^ and Zn^2+^ ions in the sequence Gly → Ala → Val was observed in concentrated solutions of the latter.

Mg^2+^ and Zn^2+^ ions included in the composition of the precipitates stabilize the amorphous phase up to 400 °C, after which they stimulate its transformation into Mg, Zn substituted β-TCP without intermediate phases. Both Mg^2+^ and Zn^2+^ ions preferentially substituted Ca^2+^ ions at the octahedral sites of (Mg, Zn)-β-TCP, and in some cases, fully displaced the Ca^2+^ ions.

The concentration of Mg and Zn in human bones vary up to 2.5 mol% Mg and up to 0.16 mol% Zn [36] which is lower than quantitative ranges of Mg (5.86–8.09 mol%) and Zn (0.71–2.82 mol%) that were prepared in ceramic powders during this study. Used in the composition of a biomaterial for bone remodeling, they can act as a reservoir for Mg and Zn ions to maintain their physiological extracellular concentrations. Our earlier research on the behavior of calcium phosphates substituted with Mg-only or Zn-only demonstrated that they did not alter the materials’ biocompatibility over a broad concentration range [18], and that when they came into contact with various simulated body fluids, the ions were gradually released into the solutions [37,38].

## Figures and Tables

**Table 1:** Thermodynamic formation constants included in the used database.

Reaction	log*K*	Source
Mg_3_(PO_4_)_2_·8H_2_O = 3Mg^2+^ + 2PO_4_^3−^ + 8H_2_O	−25.20	[27]
Mg_3_(PO_4_)_2_.22H_2_O = 3Mg^2+^ + 2PO_4_^3−^ + 22H_2_O	−23.30	[27]
KMgPO_4_·6H_2_O = Mg^2+^ + K^+^ + PO_4_^3−^ + 6H_2_O	−10.62	[27]
H^+^ + Ala^−^ = H(Ala)	9.72	[28]
2H^+^ + Ala^−^ = H_2_(Ala)+	12.05	[28]
H^+^ + Val^−^ = H(Val)	9.54	[28]
2H^+^ + Val^−^ = H_2_(Val)^+^	11.82	[28]
Ca^2+^ + Ala^−^ = Ca(Ala)^+^	1.24	[28]
Mg^2+^ + Ala^−^ = Mg(Ala)^+^	1.96	[28]
Zn^2+^ + Ala^−^ = Zn(Ala)^+^	5.21	[28]
Ca^2+^ + Val^−^ = Ca(Val)^+^	1.02	This study
Mg^2+^ + Val^−^ = Mg(Val)^+^	1.72	This study
Zn^2+^ + Val^−^ = Zn(Val)^+^	5.00	[28]

**Table 2:** Composition of the conventional (SBFc), modified (solutions 1, 2 and 3) simulated body fluids and of the buffer solution, g L^−1^.

Components	SBFc [31]	Solution 1	Solution 2	Solution 3	Buffer Solution
Na^+^	3.26	3.26	3.26	3.26	2.30
K^+^	0.12	19.8	0.12	0.12	
Mg^2+^	0.04	0.04	1.41	0.04	
Ca^2+^	0.10		14.7	0.10	
Zn^2+^				0.86	
Cl^−^	5.07	5.07	26.2	6.03	3.46
SO_4_^2−^	0.05	0.05	0.05	0.05	
HCO_3_^−^	0.26	0.78			
HPO_4_^2−^	0.10	24.1			
Gly/Ala/Val					
Series A		7.5/7.5/7.5	7.5/7.5/7.5	7.5/7.5/7.5	7.5/7.5/7.5
Series B		220/142/60	220/142/60	220/142/60	220/142/60
pH	7.2–7.4	8.0–8.2	8.0–8.2	6.5	8.0

**Table 3:** Composition of the obtained calcium phosphates.

Sample	C_Mg_	C_Zn_	(Ca^2+^ + Mg^2+^ + Zn^2+^)/P
	mol%		Molar Ratio
Series A			
CPGly7	7.63	2.61	1.54
CPAla7	7.73	2.72	1.56
CPVal7	7.91	2.82	1.56
Series B			
CPGly220	5.86	0.71	1.47
CPAla142	6.37	1.73	1.54
CPVal60	8.09	2.63	1.56

**Table 4:** Specific surface area (SSA), m^2^ g^−1.^

Series A		Series B	
CPGly7	39	CPGly220	242
CPAla7	37	CPAla142	222
CPVal7	49	CPVal60	48

**Table 5:** Unit cell parameters, mean coherent domain size (mean crystallite size), unit cell volume and % of impurity phase for samples of β-TCP synthesized from different amino acid solutions.

Sample	C_Mg_ + C_Zn_ mol,%	*a* [Å]	*c* [Å]	Mean Size [nm]	V, [Å^3^]	Ca_2_P_2_O_7_ wt%
β—TCP [34]		10.4352 (2)	37.4029 (5)		3482	
Series A						
CPGly7	10.3	10.3240 (1)	37.2646 (3)	287 (1)	3439	4.34
CPAla7	10.5	10.3234 (1)	37.2623 (6)	280 (2)	3439	3.58
CPVal7	10.7	10.3211 (2)	37.3006 (8)	322 (1)	3441	5.23
Series B						
CPGly220	6.57	10.3677 (1)	37.2023 (3)	372 (2)	3463	3.87
CPAla142	8.10	10.3503 (1)	37.1326 (3)	309 (5)	3445	3.37
CPVal60	10.7	10.3238 (1)	37.2624 (6)	276 (2)	3439	1.44

**Table 6:** Occupancies of Ca(4) and Ca(5) in Mg, Zn substituted β-TCP.

Samples	Ca(4) Position			Ca(5) Position		
	Ca	Mg	Zn	Ca	Mg	Zn
Series A						
CPGly7	0.45	0.05	0.00	0.30	0.66	0.05
CPAla7	0.29	0.07	0.14	0.08	0.76	0.15
CPVal7	0.33	0.08	0.09	0.00	0.86	0.14
Series B						
CPGly220	0.37	0.10	0.02	0.24	0.62	0.13
CPAla142	0.33	0.13	0.04	0.16	0.65	0.19
CPVal60	0.43	0.07	0.00	0.06	0.76	0.18

## Data Availability

The data presented in this study are available in this article and in Supplementary Materials.

## Supplementary Materials

The following supporting information can be downloaded at: https://www.mdpi.com/article/10.3390/ma16206638/s1, Table S1. Thermodynamic formation constants of the aqueous species used in calculation [39,40,41]. Table S2. Thermodynamic formation constants of the solids used in calculation [39,40,42,43,44,45,46,47].
