# Supplementary material for: Mg, Zn Substituted Calcium Phosphates—Thermodynamic Modeling, Biomimetic Synthesis in the Presence of Low-Weight Amino Acids and High Temperature Properties
Source: Materials (Basel). 2023 Oct 11;16(20):6638. doi: 10.3390/ma16206638 (PMC10608216; doi:10.3390/ma16206638)
Supplement: Supplementary file 1 [file materials-16-06638-s001.zip › materials-2643260-supplementary.pdf]

Supplementary material

Table S1 Thermodynamic formation constants of the aqueous species used in calculation

**Reaction**	**log*K***	**Source of log*K***
$H_2O \rightleftharpoons OH^- + H^+$	–13.997	NIST46.4
$H^+ + CO_3^{2-} \rightleftharpoons HCO_3^-$	10.329	PlumBus82
$2H^+ + CO_3^{2-} \rightleftharpoons H_2CO_3$	16.681	NIST46.7
$H^+ + SO_4^{2-} \rightleftharpoons HSO_4^-$	1.99	NIST46.7
$H^+ + PO_4^{3-} \rightleftharpoons HPO_4^{2-}$	12.375	NIST46.7
$2H^+ + PO_4^{3-} \rightleftharpoons H_2PO_4^-$	19.573	NIST46.7
$3H^+ + PO_4^{3-} \rightleftharpoons H_3PO_4$	21.721	NIST46.7
H^+ + glycine$^-$ $\rightleftharpoons$ H(glycine)	9.778	NIST46.6
$2H^+$ + glycine$^-$ $\rightleftharpoons$ H_2(glycine)$^+$	12.128	NIST46.6
$Na^+ + OH^- \rightleftharpoons NaOH$	0.1	NIST46.7
$Na^+ + Cl^- \rightleftharpoons NaCl$	–0.3	NIST46.7
$Na^+ + CO_3^{2-} \rightleftharpoons NaCO_3^-$	1.27	NIST46.7
$Na^+ + H^+ + CO_3^{2-} \rightleftharpoons NaHCO_3$	10.079	NIST46.7
$Na^+ + H^+ + PO_4^{3-} \rightleftharpoons NaHPO_4^-$	13.445	NIST46.7
$Na^+ + PO_4^{3-} + 2H^+ \rightleftharpoons NaH_2PO_4$	19.873	NIST46.7
$Na^+ + PO_4^{3-} \rightleftharpoons NaPO_4^{2-}$	1.43	NIST46.7
$2Na^+ + PO_4^{3-} \rightleftharpoons Na_2PO_4^-$	1.16	NIST46.7
$2Na^+ + PO_4^{3-} + H^+ \rightleftharpoons Na_2HPO_4$	13.32	NIST46.7
$K^+ + H_2O \rightleftharpoons KOH + H^+$	–13.757	NIST46.7
$K^+ + Cl^- \rightleftharpoons KCl$	–0.3	NIST46.7
$K^+ + SO_4^{2-} \rightleftharpoons KSO_4^-$	0.85	NIST46.7
$K^+ + H^+ + PO_4^{3-} \rightleftharpoons KHPO_4^-$	13.255	NIST46.7
$K^+ + PO_4^{3-} \rightleftharpoons KPO_4^{2-}$	1.43	NIST46.7
$2K^+ + PO_4^{3-} \rightleftharpoons K_2PO_4^-$	0.83	NIST46.7
$K^+ + PO_4^{3-} + 2H^+ \rightleftharpoons KH_2PO_4$	19.873	NIST46.7
$2K^+ + PO_4^{3-} + H^+ \rightleftharpoons K_2HPO_4$	13.44	NIST46.7
$Mg^{2+} + H_2O \rightleftharpoons MgOH^+ + H^+$	–11.397	NIST46.7
$Mg^{2+} + Cl^- \rightleftharpoons MgCl^+$	0.6	NIST46.7
$Mg^{2+} + CO_3^{2-} \rightleftharpoons MgCO_3$	2.92	NIST46.7
$Mg^{2+} + H^+ + CO_3^{2+} \rightleftharpoons MgHCO_3^+$	11.339	NIST46.7
$Mg^{2+} + SO_4^{2-} \rightleftharpoons MgSO_4$	2.26	NIST46.7

$Mg^{2+} + PO_4^{3-} \rightleftharpoons MgPO_4^-$	4.654	Turner et al.1981
$Mg^{2+} + H^+ + PO_4^{3-} \rightleftharpoons MgHPO_4$	15.175	NIST46.7
$Mg^{2+} + 2H^+ + PO_4^{3-} \rightleftharpoons MgH_2PO_4^+$	21.2561	NIST46.7
Mg^{2+} + glycine$^-$ $\rightleftharpoons$ Mg(glycine)$^+$	2.08	NIST46.6
$Ca^{2+} + H_2O \rightleftharpoons CaOH^+ + H^+$	–12.697	NIST46.7
$Ca^{2+} + Cl^- \rightleftharpoons CaCl^+$	0.4	NIST46.7
$Ca^{2+} + H^+ + CO_3^{2-} \rightleftharpoons CaHCO_3^+$	11.599	NIST46.3
$Ca^{2+} + CO_3^{2-} \rightleftharpoons CaCO_3$	3.2	NIST46.3
$Ca^{2+} + SO_4^{2-} \rightleftharpoons CaSO_4$	2.36	NIST46.7
$Ca^{2+} + PO_4^{3-} \rightleftharpoons CaPO_4^-$	6.46	Turner et al.1981
$Ca^{2+} + H^+ + PO_4^{3-} \rightleftharpoons CaHPO^4$	15.035	NIST46.7
$Ca^{2+} + 2H^+ + PO_4^{3-} \rightleftharpoons CaH_2PO_4^+$	20.923	NIST46.7
Ca^{2+} + glycine$^-$ $\rightleftharpoons$ Ca(glycine)$^+$	1.39	NIST46.6
$Zn^{2+} + H_2O \rightleftharpoons ZnOH^+ + H^+$	–8.997	NIST46.7
$Zn^{2+} + 2H_2O \rightleftharpoons Zn(OH)_2 + 2H^+$	–16.894	NIST46.7
$Zn^{2+} + 3H_2O \rightleftharpoons Zn(OH)_3^- + 3H^+$	–28.391	NIST46.7
$Zn^{2+} + 4H_2O \rightleftharpoons Zn(OH)_4^{2-} + 4H^+$	–41.188	NIST46.7
$2Zn^{2+} + OH^- \rightleftharpoons Zn_2OH^{3+}$	–8.997	NIST46.7
$Zn^{2+} + Cl^- \rightleftharpoons ZnCl^+$	0.46	NIST46.7
$Zn^{2+} + 2Cl^- \rightleftharpoons ZnCl_2$	0.6	NIST46.3
Zn^{2+} + 3Cl- $\rightleftharpoons$ $ZnCl_3^-$	0.510	Turner et al.1981
Zn^{2+} + 4Cl- $\rightleftharpoons$ $ZnCl_4^{2-}$	0.2	Turner et al.1981
$Zn^{2+} + CO_3^{2-} \rightleftharpoons ZnCO_3$	4.76	NIST46.7
$Zn^{2+} + H^+ + CO_3^{2-} \rightleftharpoons ZnHCO_3^+$	11.829	NIST46.7
$Zn^{2+} + 2CO_3^{2-} \rightleftharpoons Zn(CO_3)_2^{2-}$	7.3	NIST46.7
$Zn^{2+} + SO_4^{2-} \rightleftharpoons ZnSO_4$	2.34	NIST46.3
$Zn^{2+} + PO_4^{3-} + H^+ \rightleftharpoons ZnHPO^4$	15.689	NIST46.8
$Zn^{2+} + PO_4^{3-} + 2H^+ \rightleftharpoons ZnH_2PO_4^+$	19.403	NIST46.8
Zn^{2+} + glycine$^-$ $\rightleftharpoons$ Zn(glycine)$^+$	5.38	NIST46.6

NIST Standard Reference Database 46, Versions, 3, 4; 6; 7, 8; Martell, A.E., Smith, RM. (eds.), NIST, Gaithersburg, USA

PlumBus82: Plummer, L.N. Busenberg, E.: The solubilities of calcite, aragonite and vaterite in CO_2–H_2O solutions between 0 and 90 °C, and an evaluation of the aqueous model for the system $CaCO_3$–CO_2–H_2O. Geochim. Cosmochim. Acta **46**, 1011–1040 (1982)

Turner et al. 1981: Turner, D.R., Whitfield. M., Dickson, A.G.:The equilibrium speciation of dissolved components in fresh water and sea water at 25 °C and 1 atm pressure. Geochim. Chosmochim. Acta **45**, 855–881 (1981)

Table S2 Thermodynamic formation constants of the solids used in calculation

Solid phase	Reaction	log K	Source of log K
Halite	$NaCl = Na^+ + Cl^-$	1.550	NIST 46.8
Natron	$Na_2CO_3 \cdot 10H_2O = 2Na^+ + CO_3^{2-} + 10H_2O$	-1.311	MTQ 3.11
Nahcolite	$NaHCO_3 = Na^+ + HCO_3^-$	-0.548	Wateq 4f
Trona	$NaHCO_3 \cdot Na_2CO_3 \cdot 2H_2O = 2H_2O + 3Na^+ + CO_3^{2-} + HCO_3^-$	-0.795	Wateq 4f
Mirabilite	$Na_2SO_4 \cdot 10H_2O = 2Na^+ + SO_4^{2-} + 10H_2O$	-1.114	MTQ 3.11
Thenardite	$Na_2SO_4 = 2Na^+ + SO_4^{2-}$	0.3217	NIST 13.1
$Mg(OH)_2$(active)	$Mg(OH)_2 + 2H^+ = Mg^{2+} + 2H_2O$	18.794	NIST 46.7
Artinite	$MgCO_3 \cdot Mg(OH)_2 \cdot 3H_2O + 2H^+ = 2Mg^{2+} + CO_3^{2-} + 5H_2O$	9.6	MTQ 3.11
Hydromagnesite	$Mg_5(CO_3)_4(OH)_2 \cdot 4H_2O + 2H^+ = 5Mg^{2+} + 4CO_3^{2-} + 6H_2O$	-8.766	MTQ 3.11
Magnesite	$MgCO_3 = Mg^{2+} + CO_3^{2-}$	-7.46	NIST 46.7
Nesquehonite	$MgCO_3 \cdot 3H_2O = Mg^{2+} + CO_3^{2-} + 3H_2O$	-4.67	NIST 46.7
Huntite	$CaMg_3(CO_3)_4 = 3Mg^{2+} + Ca^{2+} + 4CO_3^{2-}$	-29.968	MTQ 3.11
Epsomite	$MgSO_4 \cdot 7H_2O = Mg^{2+} + SO_4^{2-} + 7H_2O$	-2.1265	NIST 2.1.1
$Mg_3(PO_4)_2$	$Mg_3(PO_4)_2 = 3Mg^{2+} + 2PO_4^{3-}$	-23.28	NIST 46.7
$MgHPO_4 \cdot 3H_2O$	$MgHPO_4 \cdot 3H_2O = Mg^{2+} + H^+ + PO_4^{3-} + 3H_2O$	-18.175	NIST 46.7
Portlandite	$Ca(OH)_2 + 2H^+ = Ca^{2+} + 2H_2O$	22.804	NIST 46.7
Aragonite	$CaCO_3 = Ca^{2+} + CO_3^{2-}$	-8.3	PlumBus82
Gypsum	$CaSO_4 \cdot 2H_2O = Ca^{2+} + SO_4^{2-} + 2H_2O$	-4.61	PlumBus82
Hydroxylapatite	$Ca_5(PO_4)_3(OH) = 5Ca^{2+} + 3PO_4^{3-} + OH^-$	-58.4	Tung 88
$CaHPO_4 \cdot 2H_2O$	$CaHPO_4 \cdot 2H_2O = Ca^{2+} + H^+ + PO_4^{3-} + 2H_2O$	-18.995	NIST 46.4
$Ca_4H(PO_4)_3 \cdot 2.5H_2O$	$Ca_4H(PO_4)_3 \cdot 2.5H_2O = 4Ca^{2+} + H^+ + 3PO_4^{3-} + 2.5H_2O$	-48.30	McD 77
$Ca_3(PO_4)_2$(am)	$Ca_3(PO_4)_2 = 3Ca^{2+} + 2PO_4^{3-}$	-25.8	CHRIS 90
Whitlockite	$Ca_9Mg(HPO_4)(PO_4)_6 = 9Ca^{2+} + Mg^{2+} + H^+ + 7PO_4^{3-}$	-106.34	Hamad and Heughebaert, 86
$Zn(OH)_2$(am)	$Zn(OH)_2 + 2H^+ = Zn^{2+} + 2H_2O$	12.474	NIST 46.4
$Zn(OH)_2$(epsilon)	$Zn(OH)_2 + 2H^+ = Zn^{2+} + 2H_2O$	11.534	NIST 46.4
$Zn_2(OH)_3Cl$	$Zn_2(OH)_3Cl + 3H^+ = 2Zn^{2+} + 3H_2O + Cl^-$	15.191	NIST 46.4
$Zn_5(OH)_8Cl_2$	$Zn_5(OH)_8Cl_2 + 8H^+ = 5Zn^{2+} + 8H_2O + 2Cl^-$	38.5	MTQ 3.11

$ZnCl_2$	$ZnCl_2 = Zn^{2+} + 2Cl^-$	7.05	NIST 2.1.1
$Zn_2(OH)_2SO_4$	$Zn_2(OH)_2SO_4 + 2H^+ = 2Zn^{2+} + 2H_2O + SO_4^{2-}$	7.5	MTQ 3.11
$Zn_4(OH)_6SO_4$	$Zn_4(OH)_6SO_4 + 6H^+ = 4Zn^{2+} + 6H_2O + SO_4^{2-}$	28.4	MTQ 3.11
$Zn_3O(SO_4)_2$	$Zn_3O(SO_4)_2 + 2H^+ = 3Zn^{2+} + 2SO_4^{2-} + H_2O$	18.9135	NIST 2.1.1
Zincosite	$ZnSO_4 = Zn^{2+} + SO_4^{2-}$	3.9297	NIST 13.1
$ZnSO_4 \cdot 1H_2O$	$ZnSO_4 \cdot 1H_2O = Zn^{2+} + SO_4^{2-} + H_2O$	-0.638	NIST 2.1.1
Bianchite	$ZnSO_4 \cdot 6H_2O = Zn^{2+} + SO_4^{2-} + 6H_2O$	-1.765	MTQ 3.11
Goslarite	$ZnSO_4 \cdot 7H_2O = Zn^{2+} + SO_4^{2-} + 7H_2O$	-2.0112	NIST 2.1.1
$Zn3(PO_4)_2 \cdot 4H_2O$	$Zn_3(PO_4)_2 \cdot 4H_2O = 3Zn^{2+} + 2PO_4^{3-} + 4H_2O$	-35.42	NIST 46.7

References

NIST Standard Reference Database 46, Versions, 3, 4, 7, 8; A.E. Martell, RM. Smith (Eds.); Database 13 version 1 and NIST CRITICAL database version 2.1.1, NIST, Gaithersburg, USA.

MTQ 3.11 - US-EPA Minteq v. 3.11, US Environmental Protection Agency, CEAM, College Station Road, Athens, GH 30613-0801(1991)

Wateq 4f - Ball, J.W., Nordstrom, D.K.: WATEQ4F-User's manual, U.S., Geological Survey Open-File Report 90-129, (1991)

PlumBus82, Plummer, L.N., Busenberg, E.:. The solubilities of calcite, aragonite and vaterite in CO_2-H_2O solutions between 0 and 90ºC, and an evaluation of the aqueous model for the system $CaCO_3$-CO_2-H_2O. Geochim. Cosmochim. Acta 46**,** 1011-1040 (1982)

Tung, M.S., Eidelman, N., Sieck, B.; Brown, W.E: Octacalcium Phosphate Solubility Product from 4 to 37°**C**, Journal of Research of the National Institute of Standards and Technology. 93, 613-624 (1988)

McD 77 - McDowell, H., Gregory, T.M., Brown, W.E.: Solubility of $Ca_5(PO_4)_3OH$ in the system $Ca(OH)_2$ - H_3PO_4 - H_2O at 5, 15, 25, and 37 degree. C. *Journal* of Research of the *National* Bureau of Standards, *Section* A: Physics and Chemistry. 81A, 273-281 (1977).

CHRIS 90 - Christoffersen, M.R., Christoffersen, J., Kibalczyc, W.: Apparent solubilities of two amorphous calcium phosphates and of octacalcium phosphate in the temperature range 30-42ºC. Journal of Crystal Growth 106**,** 349-354 (1990).

Hamad and Heughebaert, 86 - Hamad M., Heughebaert J.C.: The growth of whitlockite, Journal of Crystal Growth, 79, 192–197, (1986)
